# Dr. Bowditch’s Memoir on Diaphragmatic Hernia

**Published:** 1853-06

**Authors:** 


*Dr. Bowditch's Memoir on Diaphragmatic Hernia.*—We commence in the present No. the publication of this most elaborate and learned paper, and the remainder will appear in the No. for July. Although the subject may, perhaps, have less practical interest than many others, for some of our readers, all must admire the patient research and industry with which the cases have been collected, and the analysis completed. So far as the past literature is concerned, Dr. Bowditch has exhausted the subject; and henceforth, for all time, this memoir will be referred to as embodying everything that has been contributed up to the present date, and, at the same time, it will remain as a monument of the author's zeal in behalf of medical science.

